# ALLN-177, oral enzyme therapy for hyperoxaluria

**DOI:** 10.1007/s11255-019-02098-1

**Published:** 2019-02-19

**Authors:** James E. Lingeman, Gyan Pareek, Linda Easter, Rita Pease, Danica Grujic, Lee Brettman, Craig B. Langman

**Affiliations:** 1 Department of Urology, Indiana University School of Medicine, 1801 N Senate Blvd #220, Indianapolis, IN 46202 USA; 2 Division of Urology, The Warren Alpert Medical School of Brown University, 2 Dudley Street Suite 174, Providence, RI 02905 USA; 3 Clinical and Translational Science Institute, Wake Forest University School of Medicine, 1st Floor, Meads Hall, 1 Medical Center Boulevard, Winston-Salem, NC 27106 USA; 4 Allena Pharmaceuticals, One Newton Executive Park, Suite 202, Newton, MA 02462 USA; 5 Feinberg School of Medicine, Northwestern University, Ann & Robert H. Lurie Children’s Hospital of Chicago, 225 E Chicago Avenue, Chicago, IL 60611 USA

**Keywords:** Nephrolithiasis, Oxalate, Enteric hyperoxaluria, Bariatric surgery

## Abstract

**Purpose:**

To evaluate the potential of ALLN-177, an orally administered, oxalate-specific enzyme therapy to reduce urine oxalate (UOx) excretion in patients with secondary hyperoxaluria.

**Methods:**

Sixteen male and female subjects with both hyperoxaluria and a kidney stone history were enrolled in an open-label study. Subjects continued their usual diets and therapies. During a 3-day baseline period, two 24-h (24-h) urines were collected, followed by a 4-day treatment period with ALLN-177 (7,500 units/meal, 3 × day) when three 24-h urines were collected. The primary endpoint was the change in mean 24-h UOx from baseline. Safety assessments and 24-h dietary recalls were performed throughout.

**Results:**

The study enrolled 5 subjects with enteric hyperoxaluria and 11 with idiopathic hyperoxaluria. ALLN-177 was well tolerated. Overall mean (SD) UOx decreased from 77.7 (55.9) at baseline to 63.7 (40.1) mg/24 h while on ALLN-177 therapy, with the mean reduction of 14 mg/24 h, (95% CI − 23.71, − 4.13). The calcium oxalate-relative urinary supersaturation ratio in the overall population decreased from a mean of 11.3 (5.7) to 8.8 (3.8) (− 2.8; 95% CI − 4.9, − 0.79). This difference was driven by oxalate reduction alone, but not any other urinary parameters. Mean daily dietary oxalate, calcium, and fluid intake recorded by frequent diet recall did not differ by study periods.

**Conclusion:**

ALLN-177 reduced 24-h UOx excretion, and was well tolerated. The results of this pilot study provided justification for further investigation of ALLN-177 in patients with secondary hyperoxaluria.

**Trial registration**: Clinicaltrials.gov NCT02289755.

## Introduction

Secondary hyperoxaluria, a well-known risk factor for calcium oxalate urolithiasis and oxalate nephropathy, can result from excess absorption of dietary oxalate of an unknown cause (idiopathic hyperoxaluria (IH)) or may occur in patients with enteric hyperoxaluria (EH) due to bowel disease or surgery [1, 2]. It is commonly defined as a urine oxalate (UOx) > 0.45 mmoL/1.73 m^2^/24 h, or approximately 40 mg/24 h for adults [3].

Postulated mechanisms for IH include increased intestinal absorption of oxalate, increased dietary oxalate intake, and abnormal renal tubular excretion of oxalate [4]. Although patients with IH are generally thought to have milder elevations in UOx excretion compared to patients with EH, severe hyperoxaluria and recurrent nephrolithiasis may occur in either type of hyperoxaluria [5].

EH refers to excessive UOx excretion that is a result of increased intestinal oxalate absorption due to fat malabsorption commonly seen as a complication of bariatric surgical procedures, such as the Roux-en Y gastric bypass, and in other conditions including such as cystic fibrosis (or from pancreatic insufficiency of any cause), or as a consequence of inflammatory bowel disease (Crohn’s disease), or short bowel syndrome following ileal resection for any reason [1, 6, 7].

Epidemiologic data show the association between higher UOx levels and increased risk for kidney stone formation [8]. In more than 75% of patients with kidney stones, the stones are comprised of calcium oxalate salts [9]. Kidney stone episodes are a significant risk factor for hospitalizations, surgical procedures, and in some, progressive loss of kidney function, even leading to dialysis or transplantation [10, 11]. The overall incidence and prevalence of kidney stone disease are increasing in the United States and other parts of the Western world [12, 13]. Based on data from the United States National Health and Nutritional Examination Surveys, the prevalence in the US increased from 3.2% in 1976–1980 to 5.2% in 1988–1994, and was 8.8% in the latest survey covering 2007–2010 [12]. The care of patients with kidney stones is quite costly, with direct costs in excess of 4 billion dollars in 2005 [12].

Guidelines for the management of patients with calcium oxalate kidney stones have focused on interventions to reduce hypercalciuria, if present, such as with the use of thiazide diuretics and limiting dietary salt intake, and increasing daily fluid intake. Presently, there are no approved pharmacological therapies for treating hyperoxaluria. The only management of hyperoxaluria involves recommendations for controlling and lowering the intake of dietary oxalate and increasing dietary calcium intake [14, 15] However, reducing oxalate intake is not effective in all patients, especially in those with severe hyperoxaluria. Further, remaining on a low oxalate diet for a prolonged time is quite challenging due to the presence of oxalate in many healthy foods (e.g., green vegetables, nuts, grains, fruits, chocolate, etc.) and because of its absorption-dependent increase with a high salt, high fat and low calcium content diet that is typical for many Western countries [16]. Thus, there is an unmet medical need for an effective therapy for patients with secondary hyperoxaluria and its associated complications such as kidney stones and oxalate nephropathy.

ALLN-177 is an oral crystalline formulation of the oxalate specific, microbial enzyme oxalate decarboxylase. It is developed as an oral enzyme therapy that specifically degrades oxalate along the gastrointestinal (GI) tract. Based on the pH profile of the enzyme, oxalate metabolic pathways, and experiments performed in the porcine model of diet-induced hyperoxaluria [17], the primary site of action of ALLN-177 is thought to be in the upper GI tract, primarily in the stomach. In addition, because ALLN-177 is not absorbed, and retains enzymatic activity in the distal GI tract, it may have the potential to degrade enterically eliminated oxalate secreted from the circulation into the intestine.

The effect of ALLN-177 on reducing UOx was initially shown in a placebo-controlled cross-over study in healthy volunteers with diet-induced hyperoxaluria who received ALLN-177 7500 units/meal, three times/day [18].

Herein, we describe a single-arm, open-label pilot study of ALLN-177 in subjects with secondary hyperoxaluria and a recent history of kidney stones to determine whether UOx could be reduced with daily ALLN-177 therapy. The study was conducted to inform the design of potential future studies in the patient population with secondary hyperoxaluria.

## Methods

### Study population

After IRB-approval of the study protocol, the study enrolled subjects at three centers across the United States. Male and female subjects older than 18 years of age who gave written, informed consent and who had a history of at least one kidney stone in the past 2 years, UOx ≥ 36 mg/24 h, and estimated glomerular filtration rate (eGFR) > 60 mL/min/1.73 m^2^ at screening were enrolled. Stone disease was defined as having a history of one or more of: radio-opaque stones on X-ray, history consistent with passage of a stone, stone surgery, or extracorporeal shockwave lithotripsy within the last 2 years. Subjects with primary hyperoxaluria or with 24-h urine collections at screening with > 30% difference in the creatinine/kg body weight ratio were excluded. Subjects were excluded if they had an average daily dietary oxalate intake of < 75 mg/day calculated from three diet recalls obtained during screening period, and if they used > 300 mg/day vitamin C for more than 10 days within 7 days prior to screening.

### Study design

This was an open-label pilot study with subjects serving as their own controls to obtain preliminary data on the efficacy of ALLN-177 in patients with secondary hyperoxaluria. The short duration of treatment with ALLN-177 was considered adequate for assessing the effect of ALLN-177, since therapy is oxalate specific, and its positive effect was observed immediately in both normal healthy volunteers [18] and in preclinical studies [17]. This pilot study was also intended to evaluate dietary habits and compliance with dosing and outpatient collection of 24-h urine samples, to inform future studies.

**Fig. 1:**
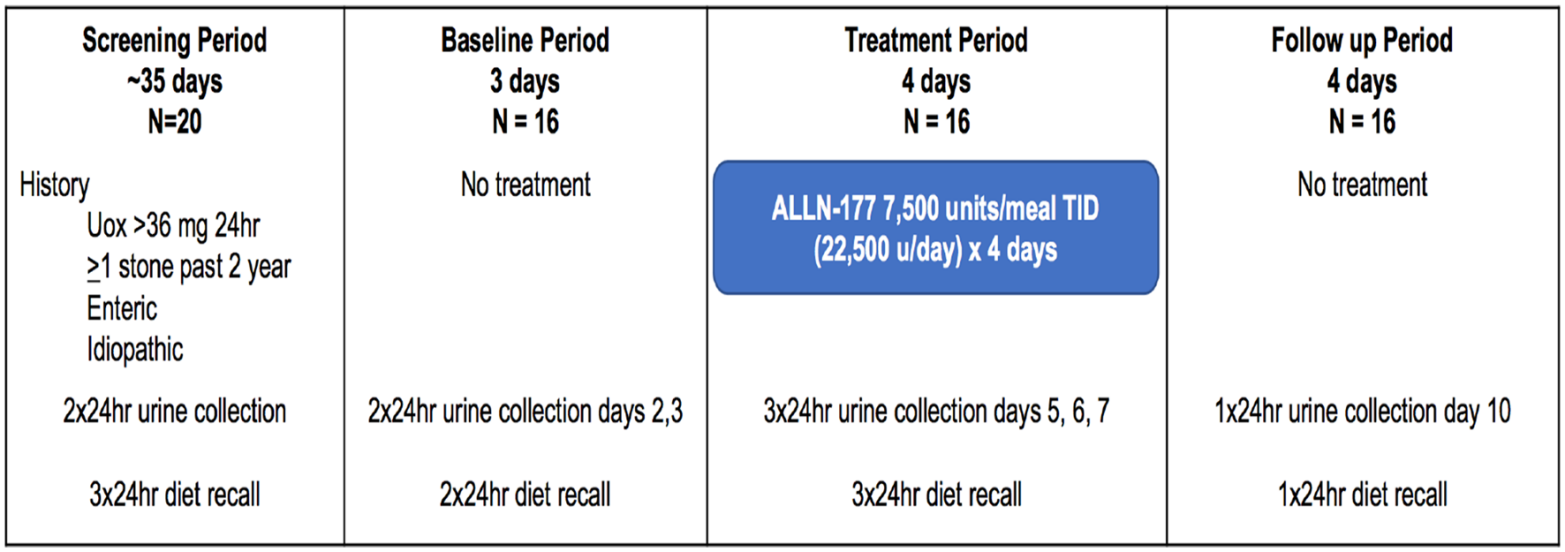
Study design: a phase 2, multicenter, outpatient, open label, single-arm study in subjects with secondary hyperoxaluria and a recent history of kidney stones. A total of eight 24-h urine collections were collected for the study. Two 24-h urines were collected during the 35-day screening period, 2 during the baseline period, 3 during the treatment period and 1 during the follow up period. A registered dietitian contacted the subject by telephone to complete a 24-h diet recall: 3 during screening, 2 during baseline, 3 during treatment, and 1 during follow up

Following a screening period of 35 days during which two 24-h urine collections were obtained to determine eligibility, subjects entered a 3-day baseline period and collected two consecutive 24-h urine samples. During the 4-day treatment period that immediately followed the baseline period, patients took ALLN-177 capsules orally with meals (7500 units/meal three times/day; total of 22,500 units daily) and collected consecutive 24-h urine on the last 3 days of the 4-day treatment period. Four days after the end of treatment, during the follow-up period, a final one 24-h urine collection was obtained (Fig. 1).

Study subjects were instructed to continue to follow their recommended usual diet throughout the study, and dietary intake was assessed nine times during the study by unannounced, telephone diet recalls conducted by a registered dietitians three dietary recalls during screening, two during baseline, three during treatment, and 1 at follow-up (Fig. 1). Throughout the study, subjects continued to use concomitant medications for modifying kidney stone risk factors, including pyridoxine (vitamin B6), thiazide diuretics (chlorthalidone, hydrochlorothiazide, amiloride with hydrochlorothiazide), citrate and calcium supplements, and allopurinol, as long as there had been no changes in these medications for at least 3 months prior to study entry. Use of vitamin C, which is metabolized into oxalate, and the use of cholestyramine, which is a bile acid sequestrant, were prohibited during the study.

Routine clinical trial safety assessments included a physical examination, adverse event (AE) assessment, standard clinical laboratory testing (hematology, serum chemistry, and urine analysis), vital sign measurements, electrocardiogram testing, and concomitant medication assessment.

### Laboratory and diet recall methods

The 24-h urine collections were analyzed at the University of Texas Southwestern, Center for Mineral Metabolism Urine Chemistry Laboratory using standard laboratory methods for urine volume, pH, calcium, citrate, chloride, magnesium, uric acid, ammonium, phosphate, potassium, creatinine and sodium, and UOx [18]. The oxalate concentration was measured using a sensitive high-performance liquid chromatography Dionex method [19], urinary calcium and magnesium levels were measured using atomic absorption spectrophotometry, and Cobas Mira plus analyser was used for measurement of creatinine, citrate and uric acid in the same laboratory [20]. Calcium-oxalate relative supersaturation was computed using the Equil2 program, and is provided as relative supersaturation ratio [21].

The 24-h diet recalls were performed by the Wake Forest Baptist Health Clinical Research Unit using a standardized script and a multiple-pass system for data collection. Participants were contacted by telephone at nine timepoints throughout the study and were asked to recall and report their dietary intake from the previous day’s 24-h period. Data were entered into the Nutrition Data System for Research (NDSR) licensed by the Nutrition Coordinating Center (NCC) at the University of Minnesota.

The NDSR software (version 2014) was used to analyze daily food and beverage intake for estimation of total daily oxalate, calcium, sodium, calories, fat, protein, carbohydrate and fluid intake collected by diet recalls [22, 23]. The NDSR is a comprehensive database providing estimated values for calcium, oxalate and other nutrients in a large number of food items.

### Statistical methods

The primary efficacy endpoint was the mean change in 24-h UOx excretion from baseline to ALLN-177 treatment [(mean baseline days 2, 6 and 3)–(mean treatment days 5, 6, and 7)]. A secondary endpoint was the percent change in 24-h UOx excretion. Summary statistics, including 95% confidence intervals (CI) of the change from baseline, were pre-specified, and paired t tests comparing treatment to baseline were derived post hoc for 24-h UOx and for the relative supersaturation ratio of calcium oxalate. Nonparametric Spearman correlation analysis was used to calculate the correlation between baseline UOx and change in UOx (Prism 7.0, Graph Pad Software, San Diego CA).

## Results

The demographic characteristics at baseline are shown in Table 1. Of the 16 patients enrolled, the majority were male, and most were Caucasian, and obese and with a preserved eGFR (> 60). Five (31%) had EH associated with bariatric surgery (three with adjustable banding, one sleeve gastrectomy, one Roux-en-Y procedure); while the remaining eleven subjects had IH. The mean (SD) length of time since onset of kidney stone disease was 13.7 (12.7) years. Concomitant medications relevant to kidney stone risk factor reduction included calcium supplements (*n* = 5), potassium citrate (*n* = 5), thiazide diuretics (*n* = 3), allopurinol (*n* = 2) and pyridoxine (*n* = 1). Compliance with the ALLN-177 treatment and with the 24-h urine collections was 100%. Diet recall compliance was > 90% in all.

The results were analyzed by all subjects and by the type of hyperoxaluria (Table 2).

**Table 1:** Baseline demographic and clinical characteristics

Characteristics	Enrolled *n* = 16
Sex—*n* (%)	
Male	9 (56.3)
Female	7 (43.8)
Age at screening—years	
Mean ± SD (median)	54.1 ± 14.5 (58)
Body mass index (BMI)—(kg/m^2^)	
Mean ± SD (median)	32.3 ± 7.7 (32.8)
Race—*n* (%)	
White/Caucasian	14 (87.5%)
Black/African American	2 (12.5%)
eGFR (mL/min/1.73 m^2^)	
Mean ± SD	85.1 ± 20.9

*eGFR* estimated glomerular filtration rat, *SD* standard deviation

**Table 2:** Summary of 24-h urine oxalate excretion (mg/24 h), percent change overall and by type of hyperoxaluria

Groups	Baseline period mean (SD)	Treatment period mean (SD)	Change from baseline mean (SD), (%)	Follow-up mean (SD)
All subjects (*n* = 16)	77.7 (55.9)	63.7 (40.1)*	− 13.9 (18.4), − 13.3	77.5 (49.2)
EH subjects (*n* = 5)	110.5 (82.9)	88.5 (57.1)	− 22.0 (26.6), − 10.4	109.8 (71.9)
IH subjects (*n* = 11)	62.7 (33.8)	52.5 (25.6)	− 10.2 (13.3), − 14.6	62.7 (28.2)

Data are shown mean (SD)

*EH* enteric hyperoxaluria, *IH* idiopathic hyperoxaluria

*p<0.05 for difference between baseline and treatment period

**Fig. 2:**
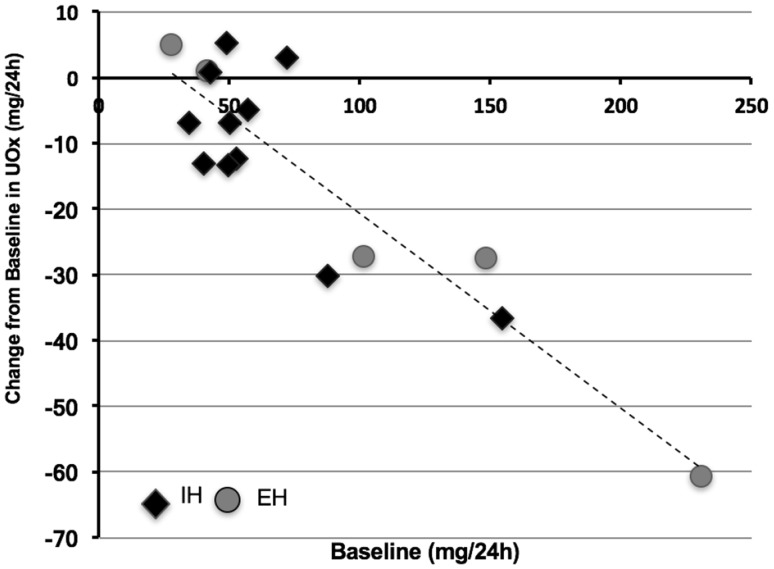
Change in mean urinary oxalate during ALLN-177 treatment compared to baseline values. Shown is a scatter plot of the baseline 24-h UOx on the X axis versus the 24-h UOx during ALLN-177 treatment on the Y axis; each diamond shape represents an individual study subject. The nonparametric Spearman’s correlation coefficient was *r* = − 0.702, *p* = 0.003

Eleven of 16 (69%) of the study subjects had a reduction in 24-h UOx, ranging between 5 and 69 mg/24 h. The magnitude of reduction was correlated with the severity of hyperoxaluria (*r*_s_ = − 0.702, *p* = 0.003). The individual changes in 24-h UOx during treatment with ALLN-177 relative to baseline are shown in Fig. 2.

The calculated mean (SD) calcium oxalate supersaturation for the overall study population decreased during treatment from 11.3 (5.7) to 8.8 (3.8) (mean change − 2.8; 95% CI − 4.9; − 0.79). The average 24-h urine pH, calcium, citrate, magnesium and other stone risk parameters remained stable throughout the study (Table 3).

**Table 3:** Summary of 24-h urine biochemistries by study period

Parameter	Baseline period mean (SD) *n* = 16	Treatment period mean (SD) *n* = 16	Follow-up mean (SD) *n* = 16
Calcium (mg/24 h)	204.2 (103.6)	205.1 (100.6)	203.6 (100.9)
Magnesium (mg/24 h)	143.8 (80.3)	141.4 (68.8)	138.3 (57.9)
Citrate (mg/24 h)	547.1 (350.7)	553.3 (351.5)	541.8 (403.3)
Creatinine (mg/24 h)	1609.3 (482.3)	1577.7 (490.9)	1588.4 (466.0)
Uric acid (mg/24 h)	572.3 (169.0)	585.2 (214.1)	566.9 (167.4)
Ammonium (meq/24 h)	39.1 (17.37)	39.5 (16.8)	41.8 (17.1)
Phosphate (mg/24 h)	1105.4 (382.7)	1033.9 (441.0)	1096.8 (359.7)
Potassium (meq/24 h)	70.2 (31.6)	68.7 (40.0)	71.6 (40.9)
Sodium (meq/24 h)	163.1 (52.8)	185 (60.6)	168.9 (62.6)
pH	5.96 (0.3)	6.029 (0.3)	5.915 (0.4)
Urine volume (L)	1.84 (0.5)	2.1 (0.6)	2.2 (0.9)
RSR CaOx	11.6 (5.7)	8.8 (3.8)*	10.8 (5.4)

Data are shown as mean (SD). RSR of calcium oxalate calculated using equil2

*CaOx* calcium oxalate, *RSR* relative supersaturation ratio

*p<0.05 for difference between baseline and treatment period

Analysis of the diet recall data revealed that subjects consumed a mean (SD) of 201.1 (121.9) mg of oxalate per day at baseline, and this did not meaningfully change during the 4 days on ALLN-177 therapy, nor during follow-up (187.4 (130) mg/d and 193.0 (139.4) mg/d, respectively). The mean (SD) daily calcium, sodium and fluid intake during treatment were 796.2 (303.4) mg, 3.56 (1.3) g, and 3.2 (1.1) L respectively, which was very similar to baseline and follow-up levels (data not shown). The mean (SD) daily calorie intake at baseline was 2301.9 (939.1) kcal and on treatment was 1989.6 (713.2), with fat comprising 37.8% and protein 14.7% of daily calories, and which did not change during the study.

ALLN-177 therapy appeared to be well tolerated. Nine subjects (56.3%) reported AEs, all of mild or moderate severity; none was a serious AE or led to study drug cessation or subject withdrawal. Only 2 (12.5%) subjects’ AEs were considered possibly related or related to study drug and were resolved within 1 day; these included abdominal distension, dyspepsia and nausea in one subject, and abdominal distension and flatulence in another subject. The evaluation of routine laboratory biochemical examinations, physical examinations, electrocardiograms, and vital signs (blood pressure and pulse rate) revealed no safety concerns (data not shown).

## Discussion

This open-label, short, pilot study was designed to evaluate whether ALLN-177, as an oral enzyme therapy taken with meals, could reduce 24-h UOx excretion in patients with histories of kidney stones and secondary hyperoxaluria and it was conducted to inform future studies.

The results of this study were comparable to the findings from preclinical studies [17], and the Phase 1 study in normal healthy volunteers with diet-induced hyperoxaluria [18]. ALLN-177 was well-tolerated. All subjects finished the study, and there were no serious or severe AEs; most were transient GI symptoms. A mean reduction in UOx of about 14 mg/24 h (13%) was observed during the 4-day treatment, and this was associated with a mean reduction in the calcium oxalate relative supersaturation ratio with no substantive changes in the other urinary parameters, suggesting that the effect of ALLN-177 is immediate and oxalate specific. The return of 24-h UOx excretion to baseline levels after discontinuation of ALLN-177 was expected, given its mechanism of action of immediate degradation of oxalate locally in the GI tract.

There appeared to be a relationship between the magnitude of reduction in 24-h Uox during treatment and the severity of hyperoxaluria at baseline (Fig. 2). Among the 11 subjects who had a reduction in UOx of > 5 mg/day while on ALLN-177, the mean baseline Uox was 91.7 mg/24 h, compared with 46.8 mg/24 h recorded among the 5 subjects without a change in UOx. This relationship was observed in both the EH and IH patients.

Endogenous production of oxalate generally does not exceed 20–25 mg/24 h, and therefore, in the absence of a primary metabolic defect, high daily UOx is most likely due to over absorption of oxalate from the diet, consistent with the current understanding of the pathophysiology of secondary hyperoxaluria [24]. Thus, patients with higher UOx levels at baseline are possibly absorbing more oxalate than patients with lower UOx, and therefore are more likely to respond to a therapy that degrades oxalate in the GI tract. Therapy was observed to be oxalate-specific since UOx returned to baseline values at the end of the 4-day follow-up period, and no other measured stone risk factors in the urine changed during treatment with ALLN-177 (Table 2).

The magnitude of reduction in UOx with ALLN-177 could be regarded as modest. However, given the pathophysiology of oxalate-related renal injury, such a reduction, if sustained over a long period of time, could potentially reduce calcium oxalate crystalluria and subsequent kidney stone formation, and other oxalate-induced renal injury [25, 26]. Epidemiologic data suggests that the lower the UOx, the lower the likelihood of being a stone former [8]. Therefore, even a modest reduction in UOx could be important therapeutically if sustained over time.

Five subjects (three with IH and two with EH) out of 16 did not have a change in UOx excretion with ALLN-177 therapy (Fig. 2). Given the variability of secondary hyperoxaluria and the design of this pilot study, the precise reasons for the individual variations of oxalate excretion and response to oral enzyme therapy in these patients is open to speculation. It may have been associated with diet and the subject’s individual variation related to stomach pH, retention time of food in the stomach, the bioavailability of oxalate from their diets, and the passage rate of ALLN-177 from the stomach to the intestine, among others.

Limitations of this study include its short duration, the small study population number, and an open-label uncontrolled design. The design was intentional and directed to inform future studies regarding endpoint selection, clinical activity, duration, study procedures and drug compliance among others, and thus a placebo control was not included in the study design. Of note, ALLN-177 is a specific enzyme therapy and its effect is immediate as shown in the initial proof of principle study of ALLN-177 in healthy volunteers [18]. Thus, we speculated that 4 days of treatment would be sufficient to show a positive effect. We also acknowledge that the colonization status of *Oxalobacter* formigenes, thought to possibly be a factor in limiting dietary oxalate absorption and reducing hyperoxaluria, was not examined [27, 28].

In the study, diet recall based on NSDR software design by Nutrition Coordinating Center University Minnesota was used to asses daily dietary changes. Dietary recalls, rather than patient maintained dietary records were collected to reduce participant burden as well as to limit participants from possibly changing their dietary behavior and thus bias recording of intakes. It is acknowledged that all forms of dietary assessment including direct observation are subject to error; however, the multiple-pass 24-h diet recall used for this study is often recommended as the method of choice for estimating dietary intake and is being utilized more frequently in research [22, 29, 30]. When conducted by trained interviewers using standardized methods, as in the current study, results tend to be more accurate than a standard 7 day food diary and importantly, present less burden to patients [30]. The NDSR was used in this study not only because of its rigorous and robust status as a research tool, but because of its uniquely comprehensive database to include complete and estimated values for calcium and oxalate in different food sources.

At present, there is no specific pharmacological therapy to reduce urinary oxalate excretion. Therapeutic agents that have been used in an attempt to lower UOx excretion in patients with hyperoxaluria have included sevelamer, a non-specific oxalate binder [31], lactic acid bacteria [32, 33] and a bacterial paste from O. formigenes that is speculated to degrade oxalate in the gut [34]. In an open-label, non-randomized study, a probiotic given to patients with EH had a transient effect in lowering UOx, and the UOx values returned to pre-treatment levels after 3 months of treatment [35]. Similarly, in a randomized, double-blind, placebo-controlled study, a probiotic (Oxadrop®) given to ten patients with calcium stones and IH had no effect on UOx excretion [35]. In another study, ten subjects with EH were enrolled in a non-randomized, open-label study of sevelamer hydrochloride for 7 days, and no change in UOx was demonstrated [31]. Lastly, in a 4-week study of patients with mild hyperoxaluria and kidney stones on a controlled metabolic low oxalate diet, the use of two different probiotics preparation did not affect Uox, but rather the diet itself had a positive effect [36].

In the absence of a more specific therapy, dietary recommendations for management include reduced dietary oxalate, salt, and protein, increased calcium intake and the use of dietary calcium supplements, and high-fluid intake. Adherence to these recommendations is quite difficult to maintain, especially in the EH population [37]. Although, dietary oxalate restriction using a controlled diet for a short time can lead to a reduction in UOx excretion in patients with mild hyperoxaluria [36], sustained effectiveness of dietary changes in patients with more severe hyperoxaluria remains to be determined. Oxalate is ubiquitous in many foods in the American Western diet [16, 36], and it is not clear that patients outside of a clinical trial can easily adhere to dietary recommendations that might reduce UOx, either due to limited availability of oxalate-free foods, lifestyle choices, or the economic costs of such specific diets. Additionally, methods of food preparation may affect the soluble oxalate content as well, such that even the same food prepared differently may yield different oxalate content [38]. Importantly, even with dietary counselling and adherence to a low oxalate dietary intake, many patients with hyperoxaluria after bariatric or other bowel surgery remain hyperoxaluric and develop stones and CKD [6, 7].

There remains an unmet medical need for an effective therapy for patients with secondary hyperoxaluria and its associated complications such as kidney stones and oxalate nephropathy. In our study, with patients with hyperoxaluria and a calcium-oxalate kidney stone history on their usual diets and standard of care including calcium supplements and thiazide diuretics, oral therapy with ALLN-177 also reduced the calcium-oxalate relative supersaturation ratio. This was driven by the reduction in UOx excretion, as other measured urine analytes did not change. Based on dietary assessment of food intake throughout the study, there were no substantive changes in dietary oxalate, calcium, sodium, or protein that would help explain the observed reduction in UOx or the relative urinary supersaturation ratio.

In conclusion, in this initial uncontrolled study in a small group of patients with secondary hyperoxaluria, oral enzyme therapy appears to be well-tolerated and reduced UOx by an average of 14 mg/24 h, without impacting other measured 24-h urine stone risk parameters. Subsequent longer-term clinical trials in subjects with secondary hyperoxaluria and kidney stones are underway.
